# Chemokines and chemokine receptors as promising targets in rheumatoid arthritis

**DOI:** 10.3389/fimmu.2023.1100869

**Published:** 2023-02-13

**Authors:** Masanori A. Murayama, Jun Shimizu, Chie Miyabe, Kazuo Yudo, Yoshishige Miyabe

**Affiliations:** ^1^ Department of Animal Models for Human Diseases, Institute of Biomedical Science, Kansai Medical University, Osaka, Japan; ^2^ Department of Immunology and Medicine, St. Marianna University School of Medicine, Kanagawa, Japan; ^3^ Department of Frontier Medicine, Institute of Medical Science, St. Marianna University School of Medicine, Kanagawa, Japan

**Keywords:** rheumatoid arthritis, chemokine, chemokine receptor, migration, leukocyte, blockade

## Abstract

Rheumatoid arthritis (RA) is an autoimmune disease that commonly causes inflammation and bone destruction in multiple joints. Inflammatory cytokines, such as IL-6 and TNF-α, play important roles in RA development and pathogenesis. Biological therapies targeting these cytokines have revolutionized RA therapy. However, approximately 50% of the patients are non-responders to these therapies. Therefore, there is an ongoing need to identify new therapeutic targets and therapies for patients with RA. In this review, we focus on the pathogenic roles of chemokines and their G-protein-coupled receptors (GPCRs) in RA. Inflamed tissues in RA, such as the synovium, highly express various chemokines to promote leukocyte migration, tightly controlled by chemokine ligand-receptor interactions. Because the inhibition of these signaling pathways results in inflammatory response regulation, chemokines and their receptors could be promising targets for RA therapy. The blockade of various chemokines and/or their receptors has yielded prospective results in preclinical trials using animal models of inflammatory arthritis. However, some of these strategies have failed in clinical trials. Nonetheless, some blockades showed promising results in early-phase clinical trials, suggesting that chemokine ligand-receptor interactions remain a promising therapeutic target for RA and other autoimmune diseases.

## 1 Introduction

Chemokines are a family of small chemotactic cytokines (approximately 8-15 kDa). Chemokine ligand-receptor interactions control leukocyte migration during inflammation, promoting migration from the circulation into the extravascular space in inflamed tissues (1, 2). Nearly 50 chemokines have been identified in mammals (3), commonly formed by four conserved cysteine residues—the first and third and the second and fourth forming disulfide bridges. Chemokines are divided into four subclasses according to the position of the first two conserved N-terminal cysteine residues: CC-chemokines (β-chemokines), having adjacent cysteine residues; CXC-chemokines (α-chemokines), having two cysteine residues separated by another amino acid; *CX_3_C*-chemokines (δ-chemokines), having two cysteine residues separated by three other amino acids; and C-chemokines (γ-chemokines), with only the second and fourth cysteine residues (4, 5). The glutamate-leucine-arginine (ELR)-positive (CXCL1, CXCL2, CXCL3, CXCL5, CXCL6, CXCL7, and CXCL8) but not ELR-negative CXC chemokines (CXCL4, CXCL4L1, CXCL9, CXCL10, and CXCL11) have three amino acid residues (Glu-Leu-Arg) before the first conserved cysteine residue. The ELR motif is important for angiogenesis (6, 7). Some chemokines are activated by matrix metalloproteinase-mediated (MMPs)-mediated proteolysis (8).

Chemokine receptors are expressed on the surface of immune cells. “Classical” chemokine receptors are G-protein-coupled transmembrane receptors (GPCRs) and induce cell migration, whereas “atypical” chemokine receptors (ACKRs) are not coupled to G proteins and regulate cell migration (9, 10). ACKRs scavenge chemokines to regulate chemokine gradients and dampen inflammation in a G protein-independent manner (3, 11, 12). Chemokine ligand-receptor interactions are presented in **Table 1** (13).

**Table 1:** The chemokines and chemokine receptors.

Name	Other names	Receptors
CC chemokine (β chemokine)		
CCL1	I-309, TCA3	CCR8
CCL2	MCP-1	CCR2, CCR4, ACKR1, ACKR2
CCL3	MIP-1α	CCR1, CCR5, ACKR2
CCL3L1	LD78β	CCR1, CCR3, CCR5, ACKR2
CCL4	MIP-1β	CCR5, ACKR2
CCL4L1	LAG-1	CCR5
CCL5	RANTES	CCR1, CCR3, CCR4, CCR5, ACKR2
CCL6	C-10, MRP-1	Unknown
CCL7	MARC, MCP-3	CCR2, CCR3, ACKR1, ACKR2
CCL8	MCP-2	Human: CCR1, CCR2, CCR3, CCR5, ACKR1, ACKR2 Mouse: CCR8, ACKR1, ACKR2
CCL9/10	MIP-1γ, MRP-2, CCF18	Unknown
CCL11	Eotaxin-1	CCR3, ACKR2
CCL12	MCP-5	CCR2
CCL13	MCP-4, NCC-1, Ckβ10	CCR2, CCR3, CCR5, ACKR1, ACKR2
CCL14	HCC-1, MCIF, Ckβ1, NCC-2, CCL	CCR1, ACKR1, ACKR2
CCL15	Leukotactun-1, HCC-2, MIP-5, NCC-3	CCR1, CCR3
CCL16	HCC-4, NCC-4, LEC (human only)	CCR1, CCR2, CCR5, ACKR1
CCL17	TARC, dendrokine, ABCD-2	CCR4, ACKR1, ACKR2
CCL18	PARC, DC-CK1, AMAC-1, Ckβ7, MIP-4	CCR8, ACKR6
CCL19	MIP-3β, ELC, Exodus-3, Ckβ11	CCR7, ACKR4
CCL20	MIP-3α, LARC, Exodus-1, Ckβ4	CCR6
CCL21	SLC, 6Ckine, Exodus-2, Ckβ9, TCA-4	CCR6, CCR7, ACKR4
CCL22	MDC, DC/β-CK	CCR4, ACKR1, ACKR2
CCL23	MPIF-1, Ckβ8, MIP-3, MPIF-1	Unknown
CCL24	Eotaxin-2, MPIF-2, Ckβ6	CCR3
CCL25	TECK, Ckβ15	CCR9, ACKR4
CCL26	Eotaxin-3, MIP-4α, IMAC, TSC-1	CCR3, CX3CR1
CCL27	CTACK, ILC, Eskine, PESKY, skinkine	CCR10
CCL28	MEC	CCR3, CCR10
CXC chemokine (α chemokine)		
CXCL1	Gro-α, GRO1, NAP-3	CXCR2, ACKR1
CXCL2	Gro-β, GRO2, MIP-2α	CXCR2, ACKR1
CXCL3	Gro-γ, GRO3, MIP-2β	CXCR2, ACKR1
CXCL4	PF-4	Unknown
CXCL4L1	PF4V1	Unknown
CXCL5	ENA-78	CXCR2, ACKR1
CXCL6	GCP-2	CXCR1, CXCR2, ACKR1
CXCL7	NAP-2, CTAPIII, β-Ta, PEP	CXCR2, ACKR1
CXCL8	IL-8, NAP-1, MDNCF, GCP-1	CXCR1, CXCR2, ACKR1
CXCL9	MIG, CRG-10	CXCR3
CXCL10	IP-10, CRG-2	CXCR3
CXCL11	I-TAC, β-R1, IP-9	CXCR3, ACKR1, ACKR4
CXCL12	SDF-1, PBSF	CXCR4, ACKR3
CXCL13	BCA-1, BLC	CXCR5, ACKR1, ACKR4
CXCL14	BRAK, bolekine	Unknown
CXCL15	Lungkine, WECHE	Unknown
CXCL16	SRPSOX	CXCR6
CXCL17	DMC, VCC-1	Unknown
CX3C chemokine (δ chemokine)		
*CX_3_CL1*	Fractalkine, Neurotactin, ABCD-3	*CX_3_CR1*
C chemokine (γ chemokine)		
XCL1	Lymphotactin α, SCM-1α, ATAC	XCR1
XCL2	Lymphotactin β, SCM-1β	XCR1

This Table is modified from Miyabe Y et al., Targeting the Chemokine System in Rheumatoid Arthritis and Vasculitis. JMA J. 2020;3(3):182-192 (13). The authors have the right to use the original Table 1 in Reference 13 and got the permission from Japan Medical Association.

The chemokine system may play a central role in rheumatoid arthritis (RA) pathogenesis. Several chemokines are highly expressed in the blood and inflammatory tissues, such as arthritic joints, of patients with RA. Furthermore, some genes encoding chemokine ligands and receptors have been reported as risk factors for RA development (14–42), and their expression is associated with clinical disease activity and severity (43–69). The regulation of immune cell recruitment into joints represents a major hallmark for therapeutic intervention, as the inhibition of the chemokine system can suppress the characteristic inflammation of RA, thereby halting its pathogenesis.

In this review, we summarize the pathogenic roles of chemokines and their receptors in RA. In addition, we provide evidence from recent human clinical trials using inhibitors of the chemokine system in RA and discuss the potential clinical benefits of chemokine blockade in patients with RA.

## 2 Rheumatoid arthritis

RA is an autoimmune disease characterized by autoantibody production, leading to the settlement of inflammatory processes with cytokine and chemokine production. This results in synovial inflammation, hyperplasia and swelling, cartilage and bone destruction and deformity, and systemic features, such as cardiovascular, pulmonary, and skeletal disorders (70).

Inflammatory cytokines, including interleukin (IL)-1, IL-6, and tumor necrosis factor (TNF)-α, play important roles in RA development. Biological agents, such as TNF-α and IL-6 inhibitors, have revolutionized RA therapies (71). However, approximately 50% of patients with RA are non-responders to these therapeutic approaches (72). Therefore, there is an ongoing need to identify novel targets and treatment strategies for RA.

Animal models of inflammatory arthritis have provided determinant information for the understanding of RA pathogenesis and development of RA therapeutics. Models such as type II collagen-induced arthritis (CIA) (73), collagen antibody-induced arthritis (CAIA) (74), K/BxN arthritogenic serum transfer model of arthritis (K/BxN) (75), and adjuvant-induced arthritis (AIA) (76) show RA-like arthritic phenotypes, including synovial hyperplasia with leukocyte infiltration and bone destruction. Furthermore, models of inflammatory arthritis and RA also show upregulated expression of chemokine ligands and their receptors in the serum, immune cells, and synovium (77–84). Thus, these animal models are useful for elucidating the pathogenic role of chemokines in RA.

### 2.1 Chemokines in RA

Various chemokines are highly expressed in the serum, synovial fluids (SFs), and synovial tissues (STs) of patients with RA compared with those of healthy donors (HD) (**Table 2**). For instance, the CC-chemokines CCL2, CCL5, CCL11, CCL13, CCL18, CCL19, CCL20, CCL22, CXC-chemokine CXCL2, CXCL5, CXCL8, CXCL9, CXCL10, CXCL11, CXCL12, CXCL13, and CXCL16 were increased in the serum and/or plasma of patients with RA compared with those of HD (43, 44, 46, 47, 54, 57, 85–90).

**Table 2:** The chemokine production in RA patients.

Source	Chemokine
Blood	CCL2, CCL5, CCL11, CCL13, CCL18-20, CCL22, CXCL2, CXCL5, CXCL8-13, CXCL16
PBMC	CCL2, CCL3, CXCL2, CX_3_CL1
T cell	CCL3, CCL4, CCL5, CXCL13
B cell	CXCL9, CXCL10
Moncyte	CCL2, CCL18, CCL19, CX_3_CL1
Macrophage	CCL25, CXCL4, CXCL7, CX_3_CL1
Dendritic cell	CCL17, CCL18, CCL19
Neutrophil	CCL3, CCL18
Endothelial cell	CCL7, CCL8, CCL14, CCL16, CCL19, CCL22
Fibroblast-like synoviocytes	CCL1-5, CCL7, CCL11, CCL13, CCL15-21, CCL25, CCL28, CXCL1-3, CXCL5, CXCL6, CXCL8-10
Chondrocyte	CCL2, CCL5, CCL13, CCL18, CCL25, CXCL1, CXCL8, CXCL10, XCL1
Osteoclast	CCL2-5, CXCL9, CXCL10, CX_3_CL1

Peripheral blood mononuclear cells (PBMCs) derived from patients with RA highly express CCL2, CCL3, CXCL2, and CX_3_CL1 compared to those derived from HD (91–93). These chemokines are differentially produced by different immune cells in patients with RA: T cells produce CCL3, CCL4, CCL5, and CXCL13 (93–96); B cells express CXCL9/10 (97); monocytes generate CCL2, CCL18, CCL19, and CX_3_CL1 (93, 98, 99); macrophages express CCL25, CXCL4, CXCL7, and CX_3_CL1 (93, 100, 101); dendritic cells (DCs) produce CCL17, CCL18, and CCL19 (102–104); and neutrophils generate CCL3 and CCL18 (103, 105, 106).

CC-chemokines are expressed in RA synovial endothelial cells (ECs) in different concentrations (high-abundance: CCL7, CCL8, CCL14, CCL16, CCL19, and CCL22; low-abundance: CCL1-3, CCL5, CCL10, CCL11, CCL12, CCL13, CCL15, CCL17, CCL18, CCL20, CCL21, CCL23, CCL24, CCL25, CCL26, CCL27, and CCL28 (107), whereas ELR^+^ CXC-chemokines (CXCL1, CXCL2, CXCL3, CXCL5, and CXCL6) are expressed in the SFs of patients with RA (108). Additionally, various CC-chemokines (CCL1, CCL2, CCL3, CCL4, CCL5, CCL7, CCL11, CCL13, CCL15, CCL17, CCL18, CCL19, CCL20, CCL21, CCL25, and CCL28), CXCL8, CXCL9, and CXCL10 are also expressed in SFs, STs, and/or fibroblast-like synoviocytes (FLSs) derived from patients with RA (86, 91, 100, 102, 109–121).

Cartilage and chondrocytes from patients with RA express CCL2, CCL5, CCL13, CCL18, CCL25, CXCL1, CXCL8, CXCL10, and XCL1 (109, 118, 122, 123). In addition, osteoclasts (OCs) and OC progenitors (OCPs) from patients with RA produce CCL2, CCL3, CCL4, CCL5, CXCL9, CXCL10, and CX_3_CL1 (124, 125).

Several chemokines (CCL3, CCL4, CCL5, CCL3L1, CCL21, CCL26, CXCL8, CXCL9, CXCL10, CXCL12, and CXCL13) have been reported as risk factors for RA development (11–25). Certain chemokines (CCL2, CCL5, CCL20, CCL23, CCL25, CXCL2, CXCL5, CXCL7, CXCL8, CXCL9, CXCL11, CXCL12, and CXCL13) are associated with disease activity and/or severity (40–58). Moreover, CCL23, CXCL9, CXCL10, CXCL11, and CXCL13 may be potential biomarkers for RA (48, 56).

### 2.2 Chemokine receptors in RA

Multiple chemokine receptors as well as chemokines contribute to RA pathogenesis (**Table 3**). Polymorphisms in CCR2, CCR5, CCR6, and CCR7-encoding genes are considered risk factors for RA development (29–42). CD4^+^ cells expressing CCR5 are increased in the blood of patients with active RA compared with that of patients with inactive RA patients and HD. Furthermore, CD4^+^ cells expressing CX_3_CR1 are decreased in patients with RA, and the CD4^+^ CD95^+^ T cell subset expressing CCR7 is associated with disease activity (63). In addition, CXCR4 and CXCL12 show higher expression in the serum and joints of patients with active RA than in those of HD and patients with RA remission. Moreover, the expression of these chemokines in the synovium has been correlated with disease score in patients with RA treated with TNF-α inhibitors (54, 55).

**Table 3:** The expression of chemokine receptors in RA patients.

Cell	Chemokine receptor
T cell	CCR2, CCR4, CCR5, CCR6, CCR7, CXCR3, CXCR4, CXCR5, CXCR6, CX_3_CR1
B cell	CCR5, CCR6, CCR7, CXCR3, CXCR4, CXCR5
Monocyte	CCR1, CCR2, CCR5, CCR9, CXCR4, CX3CR1
Macrophage	CCR7, CCR9, CXCR3
Neutrophil	CCR1, CCR5, CXCR1, CXCR2
Endothelial cell	CCR7, CCR10, CXCR2, CXCR4, CXCR5, CXCR6, CXCR7, ACKR1
Fibroblast-like synoviocytes	CCR2, CCR3, CCR5, CCR6, CCR9, CXCR2, CXCR4, CXCR6, ACKR6
Osteoclast	CCR1, CCR2, CCR4, CCR7, CCR9, CXCR2, CXCR3, CXCR4, CX_3_CR1

Chemokine receptors on T cells [CCR2, CCR4, CCR5, CCR6, CCR7, CXCR3, CXCR4, CXCR6, and CX_3_CR1 (111, 126–129)], B cells [CCR5, CCR6, CCR7, CXCR3, CXCR4, and CXCR5 (130–132)], monocytes [CCR1, CCR2, CCR5, CCR9, CXCR4, and CX_3_CR1 (33, 100, 133–137)], macrophages [CCR7, CCR9, and CXCR3 (100, 138)], and neutrophils [CCR1, CCR5, CXCR1, and CXCR2 (79, 106, 139)] were more highly expressed in patients with RA than in HD.

Stromal cells of patients with RA also express chemokine receptors. For instance, ECs express CCR7, CCR10, CXCR2, CXCR4, CXCR5, CXCR6, CXCR7, and ACKR1 (6, 140–147), whereas FLSs express CCR2, CCR3, CCR5, CCR6, CCR9, CXCR2, CXCR4, CXCR6, and ACKR6 (86, 100, 115, 148–150). OCs and OCPs express CCR1, CCR2, CCR3, CCR4, CCR7, CCR9, CXCR2, CXCR3, CXCR4, and CX_3_CR1 (124, 125).

### 2.3 The pathological function of chemokine receptors in RA

**Figure 1:**
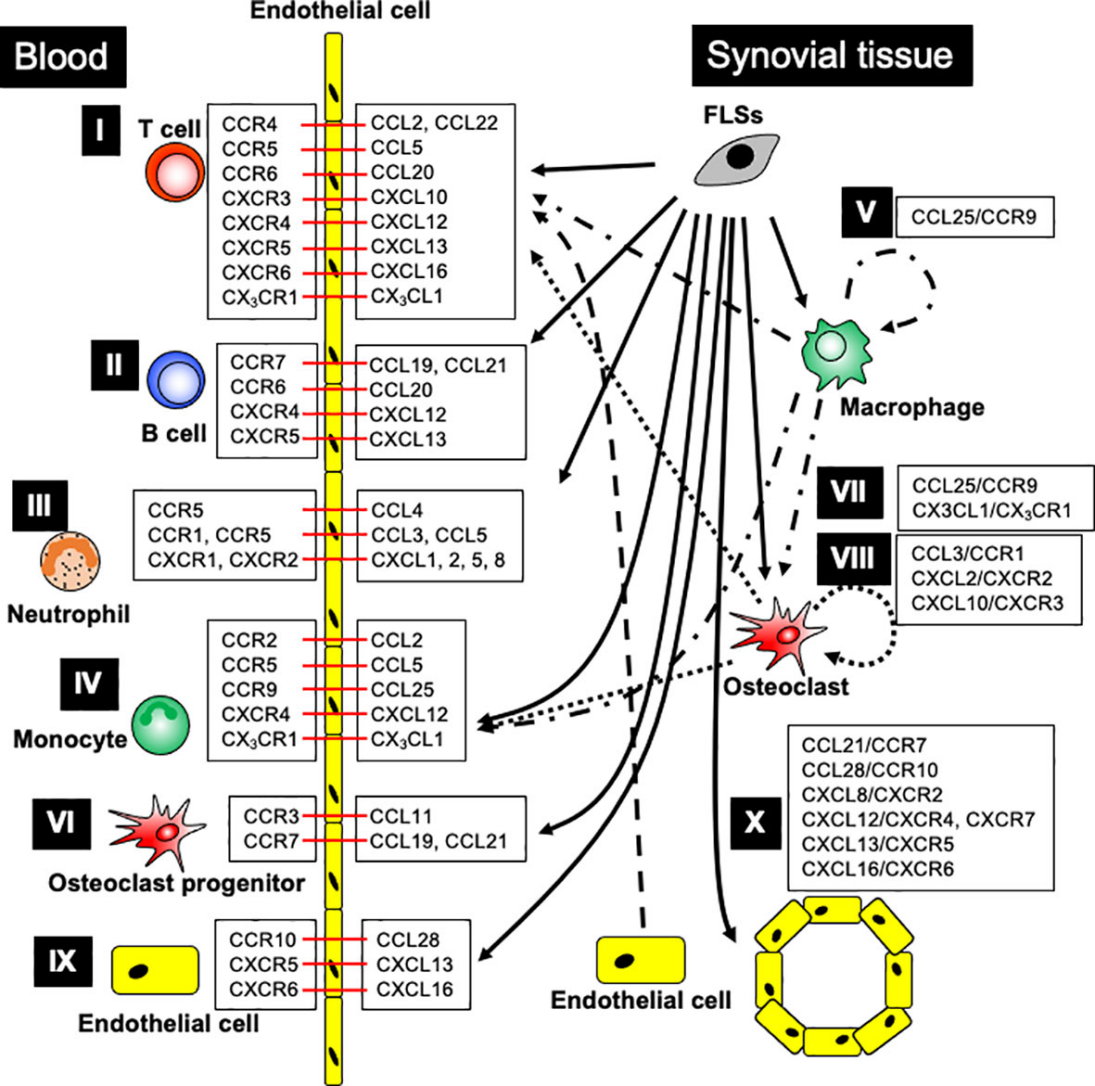
The major contribution of chemokine ligand-receptor interactions in RA patients. I. T cell recruitment: FLSs generate CCL2, CCL5, CCL20, CXCL13, and CXCL16; OCs produce CXCL10; ECs release CCL22; monocytes, macrophages, and OCs produce CX_3_CL1, promoting T cell recruitment into the arthritic joints through the indicated chemokine receptors. II. B cell recruitment: FLSs generate CCL19-21, CXCL12, and CXCL13, enhancing B cell recruitment into arthritic joints through the respective chemokine receptors. III. Neutrophil recruitment: FLSs generate CCL3-5, CXCL1, CXCL2, CXCL5, and CXCL8, leading to neutrophil recruitment into arthritic joints through the indicated chemokine receptors. IV. Monocyte recruitment: FLSs generate CCL2, CCL5, CCL25, and CXCL12; synovial macrophages produce CCL25 and CX_3_CL1; OCs produce CCL2, CCL5, and CX_3_CL1, promoting monocyte recruitment into the arthritic joints through chemokine receptor signaling. V. Synovial macrophage development: FLSs and macrophages generate CCL25, which promotes monocyte differentiation into macrophages. VI. Osteoclast progenitor recruitment: FLSs generate CCL11, CCL19, and CCL21, while ECs generate CCL19, leading to OCP recruitment into arthritic joints through the indicated chemokine receptors. VII. Osteoclast differentiation: FLSs and macrophages generate CCL25, and synovial macrophages and OCs generate CX_3_CL1, promoting osteoclast differentiation through chemokine receptors. VIII. Osteoclastogenesis: FLSs, T cells, and neutrophils generate CCL3; FLSs generate CXCL2; and OCs generate CXCL10, ensuring osteoclastogenesis through the indicated chemokine receptors. IX. Endothelial cell recruitment: FLSs generate CCL28, CXCL13, and CXCL16, stimulating endothelial cell recruitment into arthritic joints through the indicated chemokine receptors. X. Angiogenesis: FLSs generate CCL21, CCL28, CXCL8, CXCL12, CXCL13, and CXCL16, supporting angiogenesis through indicated chemokine receptors. Black arrow indicates chemokine production, and red bar shows chemokine ligand-receptor interaction.

Chemokines and their receptors control lymphocyte recruitment to inflamed joints in RA patients and animal models (**Figure 1**). In RA patients, the recruitment of T cells into the synovium is controlled by CCR4, CCR5, CXCR3, CXCR4, and CXCR6 (95, 97, 102, 126, 127, 129, 151–154). Inhibition of CCL2, CCL5, or CXCL12 suppresses Th1 cell migration *in vitro*, suggesting that these chemokines might promote Th1 cell recruitment to the RA synovium (129). CD4^+^ T cells of patients with RA treated *in vitro* with anti-CCL22 antibodies differentiate into regulatory T cells (Tregs) *via* STAT5 activation (85). In SCID mice implanted with human RA synovium, recruitment of CD4^+^ CD28^-^T cells, resembling effector memory T cells, is controlled by CCL5 and CXCL12 (127). CCR6 promotes Th17 cell recruitment into the inflamed joint in SKG arthritic mice (155), myostatin-deficient (KO) mice, TNF-α transgenic (Tg) arthritic mice (156), and chemotactic ability of Th17 cells derived from patients with RA *in vitro* model (155, 157). In addition, the CCR4 blockade suppresses Th17 cell migration to the arthritic joints in CIA mice (84). The CIA model also shows joint infiltration of CCR6^+^ type 3 innate lymphoid cells (iLC3s), which highly express IL-17A and IL-22. Furthermore, the number of CCR6^+^ iLC3s in the SF of patients with RA is correlated with disease activity (158).

Follicular helper T (Tfh) cells contribute to the formation and maintenance of germinal centers (GC). CXCR5^+^ Tfh cells are increased in the blood of patients with RA and CIA mice. Furthermore, the number of these cells correlates with the levels of clinical RA markers, such as C-reactive protein, rheumatoid factor, and erythrocyte sedimentation rate (159). In transwell experiments, the CXCL13/CXCR5 axis showed chemotactic activity in B cells of patients with RA (130). CXCR5 KO mice are resistant to CIA development; B cell-specific CXCR5 deficiency leads to mild arthritis with impaired germinal center (GC) response and antibody production, whereas T cell-specific CXCR5 deficiency promotes resistance to arthritis development by impaired GC response, antibody production, and inflammatory cytokine response (160).

CCL19, CXCL12, and CXCL13 levels in the serum of patients with RA are associated with the clinical response to rituximab (89). In addition, these chemokine levels in the SFs of patients are also correlated with both the number of CD19^+^ CD24^hi^ CD27^+^ B cells and disease activity and severity (161). The CCL20/CCR6, CXCL12/CXCR4, and CXCL13/CXCR5 axes regulate B cell migration into RA SFs (130, 161, 162), whereas the CCL19/CCR7, CCL20/CCR6, CCL21/CCR7, and CXCL12/CXCR4 axes regulate B cell recruitment into the RA synovium (130, 131).

CCL2 and CXCL8 enhance neutrophil chemotactic ability in cells from patients with RA, and CCR2 KO mice are resistant to AIA model through the suppression of CCL2/CCR2-induced neutrophil recruitment (163). CCL3 expression is associated with the neutrophil number in the SFs from patients with RA (106). Furthermore, the chemotactic activities of CCL4 and CCL5 are also correlated with the number of neutrophils in the SFs from patients with RA (94). An amino-terminal-modified methionylated form of CCL5 (Met-RANTES) antagonized the binding of CCL3 and CCL5 to their receptors CCR1 and CCR5, respectively, and the blockade inhibited arthritis in AIA rats *via* the suppression of neutrophil and macrophage migration into the joints (164).

ELR-positive CXC chemokines (CXCL1, CXCL2, CXCL3, CXCL5, CXCL6, CXCL7, and CXCL8) regulate neutrophil migration and angiogenesis *via* the receptor CXCR2 (6). CXCL5 expressed in RA SFs promotes neutrophil recruitment to EC *in vitro* (165). CXCL1 and CXCL5 induce neutrophil migration into the articular cavity of AIA mice, and chemotaxis is inhibited by the blockade of CXCR1/CXCR2 with repertaxin (79). CXCL1 and CXCL8 induce neutrophil chemotaxis *in vitro*, which is also inhibited by the blockade of CXCR1/CXCR2 and DF 2162, the later inhibiting neutrophil recruitment in zymosan-induced arthritis in mice and AIA in rats (166, 167). Furthermore, *in vitro*, the ligand for CXCR1, CXCR2, CXCL2 enhances murine neutrophil migration, and the CXCL2-neutralizing antibody inhibits migration (139). Both CXCR2 and CCR1 are expressed in mice neutrophils, and their abrogation attenuates inflammatory arthritis in K/BxN mice (168). Recent *in vivo* imaging of joints showed that CCR1 promotes neutrophil crawling on the joint endothelium, whereas CXCR2 amplifies late neutrophil recruitment and survival in the joint (169). CXCL9 blocking peptide decreases neutrophil recruitment into the joints of AIA mice (170).

*In vitro* approaches further clarified the role of some of these chemokines in RA samples. CCL2/CCR2 and CCL5/CCR5 enhance monocyte chemotaxis (171). CCL3, highly expressed in RA SFs, enhances macrophage chemotaxis (172). CCL25 induces the chemotactic activity of monocytes and differentiation into macrophages (100). CCR9 abrogation suppressed CD11b^+^ cell migration into joints in a CIA model (82). The CXCL12/CXCR4 axis promotes monocyte migration into the joints of RA ST-transplanted SCID mice (173). Furthermore, increased CX_3_CL1 expression in SFs of RA patients induced monocyte chemotaxis *via* CX_3_CR1 *in vitro* (93).

Increased OC differentiation and activity lead to bone loss and joint destruction in patients with RA. CCL3 enhanced osteoclastogenesis *via* OC migration and activation in the AIA rat model (174). CCL11/CCR3 induced OCP migration and bone resorption *in vitro* (175). CCL19 and CCL21, increased in RA SFs and serum, and their receptor CCR7, expressed in murine OCPs. These chemokines did not affect OC differentiation but promoted OC migration and increased OC resorption activity *in vitro* and *in vivo* (176). The CCL25/CCR9 axis initiates the transformation of OCPs into mature OCs *in vitro* (100). CXCL2 promotes monocyte recruitment and osteoclastogenesis in RA samples *in vitro*, as well as in mouse bone marrow-derived macrophages (90, 177). CXCL10 KO and CXCR3 KO in mice ameliorated arthritis in CAIA model by suppressing macrophage and T cell accumulation in arthritic joints. In addition, CXCL10 and CXCR3 inhibition decreased osteoclastogenic cytokine levels in the serum and spleen of CAIA (154). Furthermore, *in vitro*, CX_3_CL1/CX_3_CR1 regulates monocyte, DC, and OCP differentiation into osteoclasts (125, 178).

Several chemokines contribute to cartilage damage in arthritic joints. For instance, interferon-γ (IFN-γ) enhances CCL13 expression, inducing RA FLS proliferation in the cartilage of patients with RA *in vitro* (123). CXCL12, which induces MMP-3 production in chondrocytes *in vitro*, is also highly expressed in the SFs of patients with RA (179). CCL5 induces both MMP-1- and MMP-13-mediated collagen degradation in the SFs of patients with RA (180). In addition, CXCR4-CXCL12 signaling increased both MMP-9 and MMP-13 production in human chondrocytes *in vitro* (181).

Chemokine receptors are also expressed in stromal cells, although their functions remain unknown. Angiogenesis is determinant for RA pathogenesis, namely for synovial proliferation and pannus formation (182). CCL21, *in vitro*, induces human microvascular ECs angiogenesis and migration *via* CCR7, suggesting that the CCL21/CCR7 axis may contribute to angiogenesis in RA (140). The CXCL12/CXCR4 axis also showed angiogenic activity in RA SFs in Matrigel *in vivo* (145) and the CXCL13/CXCR5 axis facilitated EC migration and angiogenesis in CIA mice (142). CCL28 and CCR10, highly expressed in RA synovium, regulate angiogenesis by EC recruitment, and CCL28 blockade inhibits EC migration and capillary formation (141). The CXCL16/CXCR6 axis promoted chemotactic and angiogenic activity in human umbilical vein ECs (HUVEC), which is a cell line (147). The CXCL12/CXCR4 and CXCL12/CXCR7 axes promote angiogenic activity in HUVEC, contributing to RA angiogenesis. CXCR7 is also expressed on ECs in the RA synovium. Furthermore, CXCR7 blockade ameliorated arthritis in CIA mice by suppressing angiogenesis (183).

FLS-producing inflammatory cytokines and degenerative enzymes initiate synovial inflammation and joint damage in RA (184). Several chemokines (CCL11, CCL25, CXCL4, CXCL7, CXCL10, and CX_3_CL1) mediate the FLS chemotactic activity in RA *in vitro* models (82, 86, 100, 185, 186). In addition to this chemotactic activity, some chemokines (CCL2, CCL5, CCL18, CCL20, and CXCL12) increase the production of IL-6, CCL2, CXCL8, MMP-3, and COX-2 from FLS of patients with RA *in vitro* models (100, 115, 149, 150). The CX_3_CL1/CX_3_CR1 axis enhances MHVEC migration *in vitro* and angiogenesis in Matrigel *in vivo* (187). The CCL21/CCR7 axis induces VEGF and angiotensin 1 (Ang1) production in RA fibroblasts and CXCL8 and Ang1 production in macrophages (119).

## 3 Targeting the chemokine system in RA

In general, the signaling of “classical” G protein-coupled chemokine receptors is mediated by activating pertussis toxin-sensitive Gi-type G proteins. Activated G proteins regulate multiple downstream signaling cascades, such as the JAK/STAT pathway and PI3K phosphorylation (188). In contrast, signaling of “atypical” chemokine receptors is independent of G proteins and remains somewhat unclear. In this section, we provide an update on arthritis animal models and clinical trials using drugs targeting chemokines and their receptors while discussing their potential as therapeutic targets.

### 3.1 Targeting the chemokine system in animal models of inflammatory arthritis

Animal experiments are useful in the testing and development of new therapeutic agents and treatment approaches. Some chemokine ligands and receptors in KO, Tg, and naturally mutant mice are used as arthritic models (**Table 4**). For instance, CCL3 KO mice showed milder clinical and histopathological scores in the CAIA model (189), whereas *plt*/*plt* mice, a naturally occurring CCL19 and CCL20 mutant strain, also showed mild arthritis in CIA model (190). CXCL10 KO mice showed mild arthritis in CAIA model through the inhibition of macrophage and T-cell migration into the synovium (154). CXCL14 Tg mice showed exacerbated autoimmune arthritis in a CIA model, caused by an excessive immune response against type II collagen (191).

**Table 4:** The phenotypes of chemokine ligands and receptors gene-modified mice in RA models.

Gene	RA model and phenotypes
CCL3	CCL3 KO mice (C57BL/6 background) showed a mild arthritis and decreased serum amyloid P level in CAIA
CCL19, CCL21	*plt*/*plt* mice, a naturally occuring CCL19 and CCL21 mutation strain (B6N.DDD-plt/NknoJ), showed a mild arthritis in CIA
CXCL10	CXCL10 KO mice (C57BL/6 mice) showed mild arthritis, and decrease of macrophage and T cell accumulation in arthritic joints in CAIA
CXCL14	CXCL14 Tg mice (C57BL/6 background) showed severe arthritis and increased T cell and B cell response in CIA
CCR2	CCR2 KO mice (C57BL/6 background) showed decrease of neutrophil recruitment into the joints in AIA
	CCR2 KO mice (DBA/1J background) showed severe arthritis in CIA and increase of Th17 cell population, autoantibody production, and neutrohpil infiltration into joints in CIA
	CCR2 KO mice (DBA/1J, but not BALB/c background) developed arthritis than WT mice in CIA with cutaneous *M. avium* infection
	CCR2 KO mice (DBA/1J background) showed severe arthritis and elevated autoantibody production in CIA
	CCR2 KO mice (DBA/1J background) showed severe arthritis in CAIA and enhanced protease activation from monocytes and neutrophils in CAIA
	CCR2 deficiency promoted spontaneous arthritis development and neitrophil infiltration into joints in IL-1R antagonist KO mice (BALB/c background)
CCR4	CCR4 KO mice (C57BL/6 background) showed mild arthritis via inhibition of Th17 cell expansion in CIA
CCR5	CCR5 KO mice (DBA/1J background) showed mild arthritis and decrease of autoantibody production in CIA
	CCR5 KO mice (DBA/1J background) showed comparable severity with WT mice in CIA
CCR6	CCR6 KO mice (C57BL/6 background) showed mild arthritis and decrease of autoantibody production in CIA
	CCR6 KO mice (C57BL/6 background) showed comparable severity with WT mice in K/BxN
	CCR6 deficiency did not affect the arthritis development in spontaneous RA model, human TNF-α Tg mice (C57BL/6 background)
CCR7	CCR7 KO mice (C57BL/6 background) showed a completely resistance to arthritis and decrease of autoantibody production in CIA, via inhibition of DC chemotactic ability
	CCR7 KO mice (C57BL/6 background) showed mild arthritis, decrease of autoantibody production and T cell proliferation in AIA
CCR9	CCR9 KO mice (C57BL/6 background) showed mild arthritis and inhibition od CD11c-positive splenocyte migration in CIA
CXCR3	CXCR3 KO mice (C57BL/6 mice) showed mild arthritis, and decrease of macrophage and T cell accumulation in arthritic joints in CAIA
CXCR4	CXCR4 KO mice (DBA/1 background) showed resistance to arthritis in CIA
CXCR5	CXCR5 KO mice (C57BL/6 background) showed mild arthritis, decrease of autoantibody production and T cell proliferation in AIA
	CXCR5 null KO mice (C57BL/6 background) showed completely resistance to arthritis and decrease of autoantibody production, but did not affect leukocyto migration into joints in CIA
	B cell-specific CXCR5 KO mice (C57BL/6 background) showed mild arthritis and decrease GC formation in CIA
	T cell-specific CXCR5 KO mice (C57BL/6 background) showed completely resistance to arthritis and decrease GC formation in CIA
CXCR6	CXCR6 KO mice (C57BL/6 background) showed resistance to arthritis and decrease leukocyto recruitment in K/BxN
	CXCR6 KO mice (C57BL/6 background) showed resistance to arthritis and impaired cytokine polarization in T cells in CIA

CCR2 KO in the DBA/1J background exacerbated the CIA model because of the enhanced Th17 cell response and increased autoantibody production (192, 193). CCR2 deficiency in IL-1Ra KO mice enhanced neutrophil migration (194). Furthermore, CCR2 deficiency in DBA/1J caused severe arthritis in CIA with cutaneous *M. avium* infection (195). In contrast, CCR2 KO in C57BL/6 mice showed decreased neutrophil infiltration into arthritic joints in AIA model (163). CCR4, CCR6, CCR7, CCR9, CXCR5, and CXCR6 deficiency ameliorated arthritis in CIA mice by suppressing the migration of Th17 cells (CCR4), DC (CCR7), and CD11b^+^ splenocytes (CCR9) (82, 84, 160, 190, 196, 197). CCR5 KO mice showed conflicting results, with a reduced clinical score in CIA model in one study (198) and no changes in others (193). Although CCR6 KO mice were resistant to CIA model, the deficient CCR6 did not improve in an animal model of K/BxN and TNF-α Tg mice (196). In addition, CCR7 inhibition decreased autoantibody production and T cell proliferation in AIA mice (199). CXCR3 KO mice showed mild arthritis in CAIA model *via* the inhibition of both macrophage and T cell migration into the synovium (154). CXCR4-conditional KO in T cells reduced arthritic symptoms in CIA mice by inhibiting T cell migration (200). T cell- or B cell-specific CXCR5 KO mice, as well as fully CXCR5 KO mice, were resistant to both CIA and AIA models (160). CXCR6 KO mice showed resistance to K/BxN serum-induced arthritis and CIA model (147).

The blockade of a single chemokine (CCL2, CCL5, CCL24, CXCL8, CXCL9, CXCL10, and CXCL16) or chemokine receptor (CCR2, CCR5, CCR9, CXCR1, CXCR2, CXCR3, and CXCR4) demonstrated preventive and/or therapeutic effects in distinct animal models (**Table 5**). For instance, monomeric mutant CCL2, but not CCL5 mutant (^44^AANA^47^), ameliorated arthritis in AIA rats (201). Met-RANTES, which antagonizes the binding of CCL5 to CCR1 and CCR5, reduced the arthritic score and decreased macrophage infiltration into STs in CIA mice and AIA rats (83, 164). The anti-CCL5 antibody, but not the anti-CCL3 antibody, reduced the arthritic score in AIA rats (202). CCL24 blockade ameliorated arthritic symptoms in rats with AIA model (203). Anti-CXCL5 antibody ameliorated arthritis in the AIA rat model by inhibiting neutrophil migration (204). CXCL8-based decoy proteins prevented CXCR1 and CXCR2 signaling in neutrophils and ameliorated arthritis in AIA mice (205). The CXCL9 blocking peptide, which competes with CCL3 and CXCL6 binding, reduced neutrophil migration in AIA mice (170). Monoclonal bispecific antibodies against TNF-α and CXCL10 attenuated arthritis symptoms in mice by inhibiting CXCL10-mediated CD8^+^ T cell migration (206). Anti-CXCL16 antibody attenuated arthritis in CIA mice by suppressing T cell recruitment (126). Anti-CX_3_CL1 antibody decreased arthritic symptoms by inhibiting osteoclast migration into the synovium of CIA mice (207).

**Table 5:** The therapeutic effect of chemokine-targeted agents in RA models.

Target	Therapeutic effect
CCL2	Recombinant monomeric mutant CCL2 (p8A-MCP-1) protein reduced arthritic score and cytokine production in AIA rat
CCL3	Anti-CCL3 antibody did not affect arthritic score in AIA rat
CCL5	Met-RANTES reduced arthritic score in CIA mice
	Met-RANTES reduced arthritic score and macrophage infiltration into STs in AIA rat
	Recombinant CCL5 mutant (^44^AANA^47^) protein did not affect arthritic score in AIA rat
	Anti-CCL5 antibody reduced arthritic score in AIA rat
CCL24	Anti-CCL24 antibody reduced arthritic score in AIA rat
CXCL5	Anti-CXCL5 antibody reduced arthritic score and inflammatory cytokine production in AIA mice
CXCL8	CXCL8-based decoy protein reduced arthritic score and neutrophil recruitment in AIA mice
CXCL9	Antagonistic CXCL9 fragment (74–103) reduced arthritic score, neutrophil influx and cytokine production in AIA mice
CXCL10	Bispecific antibody against CXCL10 and TNF-α reduced arthritic score and CD8+ T cell migration in TNF-α Tg mice and K/BxN mice
CXCL16	Anti-CXCL16 antibody reduced arthritic score in CIA mice
CX_3_CL1	Anti-CX_3_CL1 antibody decreased arthritic symptoms by inhibition of osteoclast migration into synovium in CIA mice
CCR1	CCR1 antagonist (J-113863) reduced arthritic score, but not autoantibody production in CIA mice
CCR2	Small-molecular inhibitor of CCR2, comnined with MTX treatment reduced arthritic score and bone loss in CIA mice
	Anti-CCR2 antibody (MC) reduced arthritic score and monocyte population in blood in CIA mice
	CCR2 antagonist (MK0812) did not affect arthritic score in CAIA mice
CCR4	CCR4 antagonist (Compound 22) reduced arthritic score and decrease Th17 cells in joints in CIA mice
CCR5	CCR5 antagonist (maraviroc) decreased arthritic score and CD8+ T cell activation in CIA mice
	CCR5 antagonist (SCH-X) reduced arthritic score,but did not affect biomarkers expression in CIA monkey
	CCR5 antagonist (MCC22) did not affect arthritic score in K/B.g7 arthritic mice
CCR9	CCR9 antagonist (CCX8037) reduced arthritic score and inhibited CD11b-positive splenocyte influx into joints in CIA mice
CXCR1/ CXCR2	CXCR1/CXCR2 antagonist (SCH563705) reduced arthritic score, inflammatory cytokine production and neutrophil frequency in blood inCAIA mice
	CXCR1/CXCR2 inhibitor (DF 2162) reduced arthritic score, cytokine production and neutrophil influx in AIA rat
CXCR3	Anti-CXCR3 antibody reduced arthritic score and T cell influx into joints in adaptive transfer-induced arthritic rat
	CXCR3 antagonist (AMG487) reduced arthritic score and modulated Th17/Treg cell balance in CIA mice
	CXCR3 antagonist (SCH 546738) reduced arthritic score in CIA mice
	CXCR3 antagonist (JN-2) reduced arthritic score and cytokine production in CIA mice
CXCR4	CXCR4 antagonist, 14-mer peptide T140 reduced arthritic score and autoantibody production in CIA mice

Regarding chemokine receptors, CCR1 antagonist J-113863 decreased the arthritic score but did not affect auto-antibody production in CIA mice (208). Small-molecule inhibitors of CCR2 combined with Methotrexate (MTX) treatment reduced both the arthritic score and bone destruction *via* the suppression of OC activity in CIA mice (209). Compound 22, a CCR4 inhibitor, ameliorated arthritis by reducing Th17 cell migration into the joints of CIA mice (84). A CCR5 antagonist (maraviroc) decreased the arthritic score and CD8^+^ T cell activation in CIA mice (210); however, other CCR5 antagonists (MCC22) did not change the arthritic score in K/B.g7 arthritic mice (211). In CIA monkeys, a CCR5 antagonist (SCH-X) reduced arthritic score but did not change biomarker expression (212). CCR9 antagonist (CCX8037) reduced the arthritic score by inhibiting CD11b^+^ splenocyte recruitment into joints in CIA mice (82). The CXCR1/CXCR2 antagonist (SCH563705), but not the CCR2 antagonist (MK0812), reduced the arthritic score in CAIA mice (213). Furthermore, the blockade of CXCR1 and CXCR2 (DF 2162) ameliorated arthritis by inhibiting neutrophil migration in AIA rats (167). Anti-CXCR3 antibody reduced the arthritic scores and T cell influx into joints in adaptive transfer-induced arthritic rats (214). The CXCR3 antagonist (AMG487) contributed to the modulation of the Th17/Treg cell balance in CIA mice (215). Other CXCR3 antagonists, such as SCH 546738 and JN-2, also treated arthritis in CIA mice (216, 217). A CXCR4 antagonist (T140) reduced the arthritic score and auto-antibody production in CIA mice (218).

### 3.2 Clinical trials of chemokine-targeted therapy in human RA

Based on valuable animal research, various therapeutic agents against chemokine ligands or their receptors have been developed and tested in patients with RA (219). However, several chemokines or chemokine receptor inhibitors have failed to show positive results in clinical trials (**Table 6**). For instance, the CCL2-blocking antibody (ABN912) did not promote clinical improvements in patients with RA (220). In addition, the CCR2 antibody (MLN1202) failed at phase IIa of the clinical trial due to the reduction of monocyte levels and no changes in synovial biomarkers (221).

**Table 6:** The chemokine ligands and receptors-targeted therapy in RA patients.

Target/ Drug type	Drug name/Synonym	Released year StudyEfficacy	Study outcome	Adverse event	Inhibitory mechanism
CCL2/Antibody	ABN912/Not Available	2006Pgase Ib Not effective	There was no detectable clinical benefit of ABN912 compared with placebo.	There were no differences in the number of nature of Aes between ABN912-treated and placebo-treated patients.	The neutralizing anti-CCL2 monoclonal antibody prevents binding of the CCL2 and its receptorCCR2.
CXCL10/Antibody	MDX1100/Eldelumab, BMS-936557	2012Phase II Effective	The ACR20 response was 54% (MDX-1100 and MTX) and 17% (placebo and MTX) at weeks 12. However, ACR50, ACR70 and EULAR good responses were not significantly difference between MDX-1100- and placebo-treated patients.	51.5% of MDX-1100-treated and 30.3% of placebo-treated patients experienced AE. Serious AEs were not reported in MDX-1100- treated patients.	This neutralizing anti- CXCL10 monoclonal antibody binds to CXCL10, but not other CXCR3 ligands, CXCL9 or CXCL11.
CX_3_CL1/Antibody	E6011/Quetmolimab	2023Phase III Effective	The ACR20 response rates in E6011 200 mg and 400/200 mg were maintained 50-70% during the extension phase, and the ACR20 response rates in 100 mg were fluctuated but were maintained >45% at most time points. The ACR50 response rates in 200 mg and 400/200 mg ware maintained 25-45% during extension phase, and the ACR20 response rates in 100 mg were fluctuated but were maintained >20% at most time points. The ACR70 response rates in 400/200 mg ware maintained 15-35% during extension phase, and the ACR20 response rates in 100 mg and 200 mg were fluctuated but were maintained >10% at most time points.	The incidence of AE and TEAEs were similar across the four treatment groups (AE, 97.9% in placebo, 100.0% in E6011 200 mg, 100% in 200 mg, and 98.8% in 400/200 mg groups, and TEAE, 55.3% in placebo, 57.7% in 100 mggroup, 58.0% in 200 mg group, and 54.3% in 400/200 mg group). The incidence of serious AE was 10.7% overall.	This neutralizing anti- CX_3_CL1 monoclonal antibody prevents binding of the CX_3_CL1 and its receptor CX_3_CR1.
CCR1/Small molecule	CCX354-C/Not Available	2013Phase IINot Effective	The ACR responses were not significantly difference between placebo and CCX354-C at week 12. Only CCX354-C abundant patients in plasma showed good ACR20 response.	39% of CCX354-C (200 mg once daily)- treated, 57% of CCX354-C (100 mg twice daily) and 49% of placebo-treated patients experienced TEAR. The drug-related serious TEAE was not reported.	This orally-active small molecule is a potent and selective antagonist of CCR1.
	CP-481,715/Not Available	2010Phase IINot Effective	The ACR20 response was 34.0% (CP- 481,715 with MTX) and 47.9% (placebo with MTX) at week 6. Not significantly difference.	Not shown.	This small molecule binds CCR1 and inhibits chemotaxis activity of CCL3, CCL5, CCL7, CCL8, CCL14, CCL15 and CCL23.
	MLN3897/ AVE-9897, GSK2941266	2009Phase IIa Not effective	The ACR20 response was 35% (MLN with MTX) and 33% (placebo with MTX).	The rates of drug-related AEs (12% of both groups) and serious AEs (1% of MLN3897 and 2% of placebo) were no notable differences between MLN3897- and placebo treated patients.	This oral small molecule is CCR1 antasonist.
CCR2/Antibody	MLN1202/Plozalizumab, hu1D9	2008Phase IIa Not effective	Monocyte levels was decreased, but not synovial biomarkers (clinical response rates were similar between MLN1202 and placebo).	One patients (0.5 mg/kg MLN1202) experienced a serious AE (pericarditis) at day 42 after the last dose of study drug.	Anti-CCR2 antagonistic antibody prevents binding of the CCL2 and its receptor CCR2.
CCR5/Small molecule	Maravinoc/ Celsentri, Selzentry, UK 427857	2012Phase IIa Not effective	Maravinoc(UK-427,857) showed no significant difference in ACR20 responders (23.7%: maraviroc and 23.8: placebo) atweek 12.	55% of Maraviroc-treated patients showed TEAE such as constipation (7.8%), nausea (5.2%) and fatigue (3.9%). The serious AEs were none.	This orally bioavailable small molecule is a potent and selective antagonist of CCR5.
	SCH351125/Ancriviroc, SCH-C	2010Phase Ib Not effective	No improvement was observed by medication (3 patients did not complete, 9 patients caused serious phenotype).	20 patients received SCH351125, and 3 patients did not complete the study due to AE.	This orally bioavailable small molecule is an antagonist of CCR5.
	AZD5672/Not Available	2010Phase IIb Not Effective	The ACR response was 35% (AZD5672) and 38% (placebo).	23% of AZD5672-treated and 12% of placebo- treated patients experienced infection-related AE.	This orally bioavailable small molecule is a potent and selective antagonist of CCR5.

Animal experiments have suggested CCR5 as a good RA therapeutic candidate (198, 210–212, 222). However, reports showed that CCR5 is not determinant for RA development (223–225), and all clinical trials using CCR5 antagonists failed (226–228).

A phase II clinical trial with a CCR1 antagonist (CCX354-C) showed good efficacy in the ACR20 response in patients with abundant CCX354-C in plasma but not in those with poor CCX354-C plasma concentration. However, ACR responses did not significantly vary between placebo- and CCX354-C-treated patients (229). CCR1 antagonist (MLN3897, 10 mg, once, daily) combined with MTX had no discernible effects on the disease, despite high MLN3897 plasma concentrations and receptor occupancy of the therapeutic target (230). Another trial using a CCR1 antagonist (CP-481,715) and MTX also failed in phase II (231). CCR1 ligands, CCL3 and CCL5, can bind to other chemokine receptors, CCR3, CCR4, and CCR5 (**Table 1**). Therefore, even though CCR1 on leukocytes might be inhibited, other chemokine receptors can still promote leukocyte recruitment into inflamed joints in RA. This could explain the failures in the use of CCR1 as a therapeutic target.

In contrast, the combination of CXCL10 blocking antibody (MDX-1100) and MTX showed a mild therapeutic effect on the ACR20 response; however, ACR50, ACR70, and EULAR were not significantly different between the treatment and placebo groups. The frequency of adverse events (AEs) in MDX-1100-treated patients was higher than that in placebo-treated patients; but any MDX-1100-treated patients experienced serious AEs (232). Phase III of the clinical trial using MDX-1100 has not yet been launched.

The clinical trial using CX3CL1 blocking antibody (E6011, 200-400 mg) was effective for ACR20, ACR50 and ACR70 responses in RA patients with an inadequate response to MTX. The incidence of AEs and treatment-related AEs (TEAEs) were similar across the four treatment groups (placebo, E6011 100 mg, 200 mg, and 400/200 mg groups). Nonetheless, the incidence of serious AEs was similar between E6011- and placebo-treated patients. AEs such as nasopharyngitis, upper respiratory tract infections, bronchitis, pharyngitis, stomatitis, and back pain occurred in over 5% of the overall patients (233). However E6011 was no clear benefit in the ACR20 response rate was observed in RA patients with an inadequate response to biological DMARDs (234).

Chemokine-targeted therapy encompassed several AEs; however, the overall incidence of AEs was 40-50%, and the incidence of serious AE was 0-5% in chemokine-targeted therapies (**Table 6**). These numbers increased to an AE incidence of 60-80% and serious AEs of 5-25% in patients treated with anti-IL-6R antibody, tocilizumab (235–237). Furthermore, AE incidence was 50-70%, and serious AE incidence was 5-10% in trials using anti-TNF-α antibody, infliximab (238–240). These clinical findings suggest that chemokine-targeted therapy is safer for patients with RA than cytokine-targeted therapy.

In addition to the above-mentioned blockade agents, other inhibitors of chemokine ligands or their receptors have demonstrated therapeutic effects on arthritis in RA models. Thus, these chemokine ligands and respective receptors may be promising targets for new RA therapies.

## 4 Conclusion

In this review, we summarize the functional roles of chemokine ligand–receptor interactions in arthritic joints of animal models and RA patients. Although several inhibitors of chemokines and/or their receptors have shown therapeutic effects in animal models of arthritis and clinical trials of patients with RA, limited therapeutic effects have been reported, suggesting that chemokine-targeted therapy still requires improvement. In targeting chemokine receptors, the choice of the most relevant receptor and ensuring high receptor occupancy at all times might be the key to therapeutic effects. In addition, inhibition of a single chemokine alone may not be sufficient to completely suppress leukocyte migration due to the functional overlap between chemokine systems. Therefore, the combined targeting of multiple chemokines and/or their receptors may be a more effective approach for human RA. Our previous study in animal models demonstrated that broadly cross-reactive chemokine-blocking antibodies for CXCR2 ligands dramatically ameliorated inflammatory arthritis compared with inhibition with antibodies against a single chemokine (241).

Further understanding of the importance of different chemokines at different stages of RA is required for the development of drugs that effectively target the system. We have previously developed an *in vivo* imaging technique to fully dissect the functional roles of chemokines and their receptors in inflamed joints in animal models (242). Interestingly, CXCR2 and ACKR1 are required for neutrophil apoptosis in the joint space, whereas the classical C5aR1 and atypical C5a and C5aR2 receptors are required for neutrophil apoptosis in the joint (146, 169). Altogether, the development of effective inhibitors of chemokines and their receptors has untapped therapeutic potential in RA.

## Author contributions

MM and YM conceived the manuscript; MM prepared the draft manuscript; MM, JS, CM, KY and YM approved the published version of the manuscript.
